# Editorial: Clinical Integration of Artificial Intelligence in Spine Surgery: Stepping in a new Frontier

**DOI:** 10.3389/fsurg.2023.1351643

**Published:** 2023-12-21

**Authors:** Enrico Gallazzi, Giovanni Andrea La Maida, Federico Cabitza

**Affiliations:** ^1^ U.O.C. Patologia Vertebrale e Scoliosi, ASST Gatano Pini - CTO, Milano, Italy; ^2^ Department of Informatics, Systems and Communication, University of Milano-Bicocca, Milano, Italy; ^3^ IRCCS Ospedale Galeazzi - Sant'Ambrogio, Milano, Italy

**Keywords:** spine surgery, cervical spondylotic myelopathy, artificial intelligence, machine learning, deep neural network

**Editorial on the Research Topic** Clinical Integration of Artificial Intelligence in Spine Surgery: Stepping in a new Frontier

The application of artificial intelligence (AI) in healthcare has expanded rapidly in recent years. In spine surgery specifically, there is growing interest in leveraging AI technologies to improve diagnosis, surgical planning, intraoperative guidance, and postoperative care.

This was reflected by the huge increase in the number of published papers that explore potential AI application in Spinal pathologies in the last few years. Nonetheless, to ensure the highest possible quality of research, it is not enough to merely applying AI to medical data: studies should indeed be aimed at developing tools that medical staff “can use in their daily practice to improve the appropriateness, safety and effectiveness of their decisions, and ultimately the health outcomes of their Patients” (1).

Following this principle, this special issue focuses on translating AI innovations into clinical practice within the field of spine surgery. The aims of this special issue are twofold. First, it brings together high-quality studies that demonstrate the reliability, robustness, and clinical utility of AI systems for spine surgery. Second, it provides a forum to discuss both the promises and challenges of integrating AI into routine surgical workflows. Meeting these aims will accelerate the adoption of impactful AI tools that ultimately benefit patient care.

The scope of the special issue spans the continuum of spine surgery, from diagnosis to postoperative follow-up. Contributions apply AI techniques including machine learning and deep learning for tasks such as image analysis, patient risk stratification, surgical planning, intraoperative navigation, and outcome prediction. Importantly, the selected studies provide evidence of clinical viability based on the accuracy of ground truth labels, generalization to new data, and measurable improvements in surgical decision-making or patient outcomes.

Several, high quality paper met the Editorial standard for this Special Issue and provided extremely interesting and innovative application of AI in different Spinal pathologies.

Two papers investigated potential clinical application of AI in Cervical Spondylothic Myelopathy (CSM). CSM is the leading causes of paraparesis in Adult Population, with an estimated prevalence of 2%–4% (2). The most effective treatment in reducing or halting disease progression is decompressive surgery; however, huge heterogenicity in clinical presentation and surgical decision-making is reported (3), thus making research in this field extremely important. In the paper by Zhou et al., the Authors demonstrates the ability of unsupervised machine learning algorithms to identify two clinically distinct clusters of patients that underwent Anterior Decompression and Fusion for CSM, on the basis of preoperative characteristics. While the two clusters differed in comorbidities, risk factors and disease severity, the postoperative outcomes were similar between the two groups, hence suggesting that more at risk, more ill patients could benefit most from surgery. This data-driven approach to patient phenotyping could serve as a foundation for more personalized, data-driven treatment paradigms (Zhou et al.). The second paper by Park et al., focuses on modeling expert therapeutic decisions for CSM using ensemble machine learning. Their models accurately classified treatment options (conservative, anterior approach, posterior approach) based on a range of clinical and radiographic variables. Interestingly, the most important variables used by the AI to distinguish between conservative versus surgical group were the modified Japanese Orthopedic Association score and Central Motor Conduction Time, thus giving less importance to radiographic parameters; on the contrary, number of levels involved was the most important parameter to distinguish between anterior and posterior approach. The models' high predictive performance suggests a promising role for AI-assisted surgical planning in this patient population, potentially simplifying the current, “classical” treatment algorithms (Park et al.). Both those studies offer an interesting framework for the use of AI in CSM, with evident and relevant clinical implications.

The study by Mekhael et al., focuses instead on Adult Spinal Deformities (ASD). ASD is a complex disease, with a huge burden on quality of life; only a small fraction of patients with ASD require surgery, and correct identification of those patients is extremely important given the extremely high frequency of surgical complications. In their paper, they used an innovative machine learning (ML) approach to predict Health related Quality of Life (HRQoL) in ASD, combining radiological parameters with kinematics parameters derived from 3D movement analysis (3DMA) (Mekhael et al.); in their ML simulation, they found that the combination of radiological and kinematic parameters predicted HRQoL with greater accuracy than stand alone classical radiographic analysis. Moreover, they showed how 3DMA was a better predictor of HRQoL after surgery. Overall, their paper offers a new perspective on the clinical evaluation of patients with ASD: the addition of 3DMA enhanced by AI could be extremely helpful in better stratifying ASD patients, allowing for a better selection of patients that will actually benefit from surgery.

In the fourth paper of this collection, Bassani et al. tested a deep learning approach to classify endplate lesions on sagittal MRI (Bassani et al.). Correctly identifying endplate lesion could be important in screening patients with degenerative lumbar disease. Their algorithm showed a high accuracy in classifying those lesions: thus, AI could be used to perform a repetitive task (classification), potentially reducing Doctors screen time.

Finally, the bibliometric analysis by Fan et al. summarizes hot topics and trends in research on lumbar spondylolisthesis, as determined through text mining and machine learning. The authors found that surgical management is the major topic in lumbar spondylolisthesis research, followed by radiographic assessment and epidemiology. The authors also identified minimally invasive techniques as a recent hotspot in lumbar spondylolisthesis research. Beyond characterizing the recent literature, these scientometric insights can inform future research directions to advance the field (Fan et al.).

Together, these articles demonstrate innovative applications of AI that hints at the potential to profoundly shape clinical practice in spine surgery. AI-powered systems can be used to identify heterogeneous patient populations, classify expert-level therapeutic decisions, and identify research topics and hotspot trends. As AI technology continues to develop, we can expect to see even more innovative and transformative applications of AI in spine surgery. This special issue provides a rigorous forum to evaluate AI systems for integration into real-world surgical settings. It is Editors' opinion that, through collaborative efforts across clinical, technical, and industry partners, AI technologies can be thoughtfully implemented to improve spine patient care.
